# Utilizing Roche Cedex Bio analyzer for in process monitoring in biotech production

**DOI:** 10.1186/1753-6561-5-S8-P106

**Published:** 2011-11-22

**Authors:** Dörthe Druhmann, Sabrina Reinhard, Felizitas Schwarz, Christina Schaaf, Katrin Greisl, Tim Nötzel

**Affiliations:** 1 Roche Diagnostics GmbH, Pharma Biotech Penzberg

## Introduction

One task during development and control of bioprocesses used for production of recombinant proteins is to deliver fast, accurate and reliable analytical process data.

Monitoring of nutrients and metabolites in animal or bacterial cell culture is essential to avoid limitations or toxic accumulation during fermentations that can effect cell growth, viability, product yield and quality. Within certain fermentation processes low levels of nutrients trigger in-process actions such as feed. Other parameters are used to track culture reproducibility and to gain process understanding for process development and validation.

Typical parameters in mammalian cell culture are glucose, lactate, glutamine, glutamate, ammonia, sodium and potassium. Therefore it is common to use multi-analyzer systems based on enzymatic-dependent biosensors and ion- selective potentiometry.

Furthermore the release of lactate dehydrogenase (LDH) into the fermentation media is used as an indicator of cell death during fermentation processes therefore a 96-well based photometric assay is used . The measurement of product titer like IgG is needed to track productivity or yield during fermentation and protein purification processes and is mostly done by HPLC methods.

One major motivation for this new Bioprocess analyzer lies within the eminent issues of enzyme membrane based analytical technology such as: short life cycles as well as poor or changing quality of the enzyme membranes, high material costs, non-linearity of measurements and in particular insufficient sensitivity and accuracy.

Additionally the laborious maintenance required to operate different analytical devices and labor intensive sample management as well as manual steps to avoid operator dependent variability needed to be reduced.

## Applying diagnostic knowledge to bioprocess care

Combining the knowledge of 25 years of diagnostic healthcare with expert knowledge in fermentation processes a new Bioprocess analyzer (Cedex Bio analyzer*) was developed. The new Cedex Bio analyzer stands out with increased sensitivity plus enlarged measurement ranges compared to currently used methods and analyzers.

An automated dilution increases the measurement ranges and reduces operator variability because no manual dilution is needed before applying the sample to the Cedex Bio analyzer.

The aim of this evaluation and method validation was to compare this new device to common analytical systems.

**Table 1:** measurement ranges current methods versus Cedex Bio

Parameter	Current methods	Cedex Bio	Cedex Bio
		w/o dilution	automated dilution
glucose [mg/L]	200 – 15.000 ^1)^	20 – 7.500	20 – 75.000
lactate [mg/L]	200 – 5.000 ^1)^	18 – 1.400	18 – 14.000
ammonia [mg/L]	3,6 – 450 ^1)^	0.48 – 12	0.48 – 480
glutamine [mg/L]	30 – 877 ^1)^	60 – 1.500	60 – 7.500
glutamate [mg/L]	30 – 882 ^1)^	15 – 1.500	15 – 7.500
sodium [mg/L]	920 – 5.006 ^2)^	460 – 5.750	460 – 5.750
potassium [mg/L]	39,1 – 978 ^2)^	39 – 1.170	39 – 1.170
LDH [U/L]	20 – 150 ^3)^	20 – 1.000	20 – 10.000
IgG [mg/L]	35 – 1.500 ^4)^	10 – 1.600	10 – 16.000

^1)^ Membrane analyzer, ^2)^ Ion selective electrode ^3)^ 96-well-plate assay, ^4)^ HPLC method

## Validation and correlation studies

**Figure 1:**
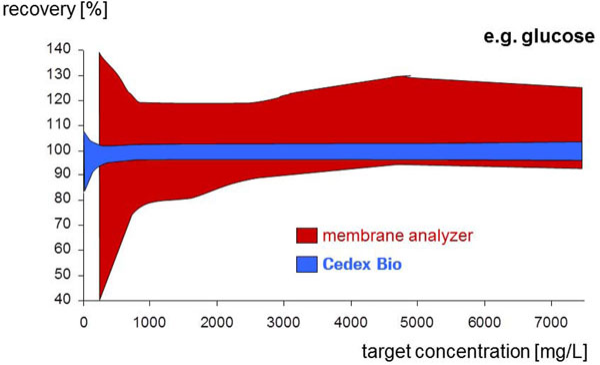
Comparison of recovery values of the Cedex Bio analyzer versus a membrane analyzer. Range of recovery of reference standards (quality control material, n ~ 250, t = 6 months)

Reference standards were analyzed at least once a day before and after each tray of samples to control the performance of the membrane analyzer. Day to day and side by side comparability as well as accuracy and linearity of the Cedex Bio analyzer are superior to the membrane analyzer as shown in Figure 1.

An increased sensitivity allows to operate nutrient limited fermentations and likewise to detect remote changes within the metabolite profile of the fermentation process.

Monitoring of fermentations processes using a membrane analyzer required laborious maintenance, a lot of calibrations and permanent control of recovery.

**Figure 2:**
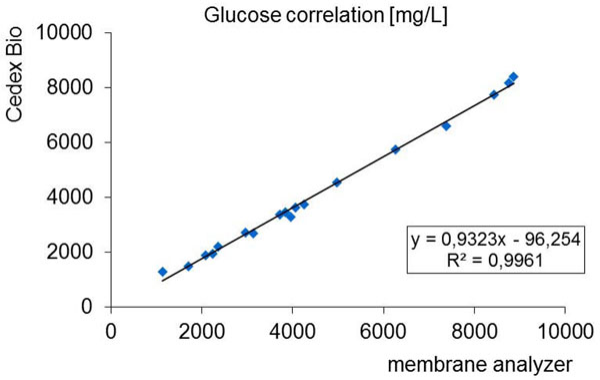
Field Data with samples from mammalian cell culture: Cedex Bio analyzer versus a membrane analyzer. The slope < 1 due to a constant overestimation of the glucose concentration by the membrane analyzer

However correlation studies conducted with samples of a fed-batch mammalian cell fermentation show that the Cedex Bio analyzer easily replaces the membrane analyzer with comparable batch data (Figure 2).

Furthermore additional parameters like LDH or IgG can be analyzed from the same sample.

## Summary

ο The Cedex Bio analyzer combines three devices to one with the possibility to measure up to 9 parameters in fermentation browth at once and from the same sample within a few minutes.

ο Automated dilution reduces operator dependent manual steps and variability.

ο Methods based on wet chemical photometric assays, ionselective electrodes and turbidity assays are easy to handle, reliable and robust.

ο The compact analyzer helps to save laboratory space and costs for different instruments.

ο Sensitive, precise and accurate analytical data allow a tight control of cell culture processes.

ο The assay portfolio will also be suitable for microbial fermentation with additional parameters like acetate or ethanol.

ο The Cedex Bio analyzer combines three devices to one with the possibility to measure up to 9 parameters in fermentation browth at once and from the same sample within a few minutes.

* CEDEX is a trademark of Roche

